# Comparative efficacy of therapeutic strategies for recurrent or refractory glioblastoma: a systematic review and network meta-analysis

**DOI:** 10.3389/fneur.2026.1896062

**Published:** 2026-08-20

**Authors:** Hao Zhang, Leilei Sun, Zhihe Yun, Xianyong Yin, Yuming Li, Jing Wu, Guangjian Dong

**Affiliations:** 1 Department of Pharmacy, The First Affiliated Hospital of Shandong First Medical University and Shandong Provincial Qianfoshan Hospital, Jinan, China; 2 Department of Medical Ultrasound, The First Affiliated Hospital of Shandong First Medical University and Shandong Provincial Qianfoshan Hospital, Jinan, China; 3 Department of Neurosurgery, The First Affiliated Hospital of Shandong First Medical University and Shandong Provincial Qianfoshan Hospital, Jinan, China

**Keywords:** bevacizumab, glioblastoma, lomustine, network meta-analysis, recurrent glioblastoma, refractory glioblastoma, systematic review

## Abstract

**Background:**

Recurrent or refractory glioblastoma has a poor prognosis, and no universally accepted standard treatment has been established. Randomized evidence is fragmented, making pairwise comparisons insufficient. This study aimed to compare available treatments using a frequentist network meta-analysis.

**Methods:**

PubMed, Web of Science, and the Cochrane Library were searched from inception to April 2026 for randomized controlled trials involving adults with recurrent or refractory glioblastoma. Prespecified outcomes were progression-free survival (PFS), overall survival (OS), and objective response rate (ORR). Hazard ratios (HRs) were used for PFS and OS, and odds ratios (ORs) for ORR. Random-effects frequentist network meta-analyses were conducted, with P-scores used as exploratory rankings. Sensitivity analyses excluded trials with fewer than 20 patients in any treatment arm. The protocol was registered with PROSPERO (CRD420261398465).

**Results:**

Twenty-four studies were included in the qualitative synthesis, of which 19 contributed to at least one network meta-analysis. Compared with lomustine, bevacizumab plus lomustine significantly improved PFS (HR = 0.57, 95% CI: 0.40–0.80), and remained significant after excluding small trials (HR = 0.58, 95% CI: 0.34–0.97). Bevacizumab plus vorinostat had the highest numerical P-score for PFS, although its effect was not statistically significant. No intervention showed a statistically significant OS advantage over bevacizumab. Rindopepimut plus bevacizumab had the most favorable numerical OS estimate (HR = 0.53, 95% CI: 0.23–1.21), but this was non-significant and derived from an EGFRvIII-positive population. No treatment significantly improved ORR versus bevacizumab, whereas nivolumab was associated with lower odds of response (OR = 0.28, 95% CI: 0.14–0.57). Excluding small trials had limited influence on PFS and OS findings but altered the ORR ranking by removing the highly ranked ERC1671-based regimen.

**Conclusion:**

Bevacizumab plus lomustine showed the clearest PFS benefit, but the absence of an OS benefit precludes interpretation as broadly superior. No treatment showed a confirmed OS advantage, and the favorable numerical signal for rindopepimut plus bevacizumab should not be generalized beyond EGFRvIII-positive patients. P-score rankings should be considered exploratory and interpreted alongside effect estimates, confidence intervals, sample sizes, molecular eligibility, safety, and treatment burden.

**Systematic review registration:**

https://www.crd.york.ac.uk/PROSPERO/view/CRD420261398465, Identifier: CRD420261398465.

## 1 Introduction

Glioblastoma is the most aggressive adult-type diffuse glioma and one of the most lethal primary malignant tumors of the central nervous system (1, 2). The 2021 World Health Organization classification emphasized the central role of integrated molecular diagnosis and defined adult-type glioblastoma within the IDH-wildtype diffuse glioma category, which is important when interpreting older clinical trials conducted before contemporary molecular classification became routine (1). Despite advances in molecular classification, surgical techniques, radiotherapy, systemic therapy, and supportive care, the prognosis of glioblastoma remains poor, and recurrence is nearly inevitable after standard initial treatment (3, 4). The current first-line approach generally includes maximal safe resection when feasible, followed by radiotherapy with concomitant and adjuvant temozolomide; tumor-treating fields may also be considered in selected patients after chemoradiotherapy (2–4). Once recurrence occurs, treatment decisions become more individualized and less evidence-standardized, and no universally accepted standard regimen has been established (5–7).

A broad range of therapeutic approaches has been investigated in recurrent or refractory glioblastoma, including alkylating chemotherapy, anti-angiogenic therapy, multi-target tyrosine kinase inhibition, immune checkpoint inhibition, re-irradiation, tumor-treating fields, and vaccine-based immunotherapy (8–14). Lomustine has frequently served as a comparator in randomized trials of recurrent glioblastoma, whereas bevacizumab is used in some clinical settings because of its radiographic response, edema control, and corticosteroid-sparing effect (6, 9–11). However, improvements in PFS or imaging response have not consistently translated into OS benefit, and the interpretation of anti-angiogenic treatment effects is further complicated by potential pseudoresponse (15, 16). Recent reviews and evidence syntheses have highlighted the heterogeneity of available therapeutic options, the variability of trial populations and endpoints, and the continued absence of a clearly preferred regimen for recurrent or refractory glioblastoma (6, 7, 17).

The evidence base is difficult to interpret using conventional pairwise meta-analysis alone. Most randomized trials evaluate one experimental strategy against a limited set of control regimens, and direct head-to-head comparisons among commonly used options remain scarce. Network meta-analysis can integrate direct and indirect evidence within a common analytical framework, allowing simultaneous comparison of multiple interventions (18, 19). Previous network meta-analyses have attempted to compare regimens for relapsing or refractory glioblastoma; however, differences in eligibility criteria, treatment-node definitions, statistical frameworks, and treatment-ranking methods may influence the interpretation of relative efficacy (20). In addition, more recent randomized evidence, including the GBM AGILE platform-trial data for regorafenib, further supports the need for an updated synthesis using a transparent and clinically interpretable framework (14).

Therefore, this study systematically reviewed randomized evidence for recurrent or refractory glioblastoma and compared therapeutic strategies using a frequentist network meta-analysis. The main efficacy outcomes were PFS, OS, and ORR. Treatment ranking was summarized with P-scores, a frequentist analogue of SUCRA, and interpreted together with effect estimates, 95% confidence intervals, network structure, and limitations of the underlying evidence (21).

## 2 Materials and methods

### 2.1 Literature search and eligibility criteria

This systematic review and network meta-analysis was conducted and reported in accordance with the PRISMA extension statement for network meta-analyses (19), with additional consideration of the PRISMA 2020 statement and the PRISMA-S extension for transparent reporting of the literature search (22, 23). The review protocol was registered with PROSPERO on 18 May 2026 (CRD420261398465). Registration was completed after the literature search had commenced but before the completion of the review and network meta-analysis (24). PubMed, Web of Science, and the Cochrane Library were systematically searched from database inception to April 2026. The search strategy combined database-specific controlled vocabulary, where applicable, and free-text terms related to the disease, recurrence status, therapeutic interventions, and randomized trial design, including glioblastoma, high-grade glioma, recurrent, refractory, relapse, progression, bevacizumab, lomustine, immunotherapy, targeted therapy, radiotherapy, tumor-treating fields, and randomized controlled trial. Search terms were adapted to the syntax and indexing structure of each database. Reference lists of eligible studies and relevant reviews were also manually screened to identify additional records. The complete search strategies, including database-specific search strings, are provided in Supplementary methods.

Randomized controlled trials were eligible if they met the following criteria: adult patients with recurrent or refractory glioblastoma, or recurrent high-grade glioma populations predominantly composed of glioblastoma; comparison of two or more active therapeutic strategies, standard treatment regimens, or placebo-controlled regimens; and availability of hazard ratios (HRs) for progression-free survival (PFS) and/or overall survival (OS), or response data allowing calculation of odds ratios (ORs) for objective response rate (ORR). Reviews, case reports, single-arm studies, observational studies, study protocols, pediatric studies, preclinical or translational studies, and conference abstracts without sufficient extractable data were excluded. When multiple reports described overlapping patient populations, the report with the most complete efficacy dataset, longest relevant follow-up, or most suitable data for the prespecified outcomes was used.

### 2.2 Outcomes and data extraction

The prespecified efficacy outcomes were PFS, OS, and ORR. PFS and OS were treated as time-to-event outcomes and summarized using HRs. ORR was defined as the proportion of patients with complete or partial response, as reported in the original trial, according to the response criteria used by each study, mainly the Macdonald criteria or Response Assessment in Neuro-Oncology (RANO) criteria (15, 25). Because response assessment criteria in glioma have evolved over time, the interpretation of radiographic response and progression was based on the definitions reported in each original study; more recent RANO 2.0 recommendations provide an updated framework for response assessment in adult gliomas but were not uniformly applicable to older trials included in this review (16).

Two reviewers independently extracted study-level information using a predefined data extraction form. Extracted variables included first author, publication year, study phase, disease setting, treatment line, intervention arms, comparator arms, sample size, available molecular information, primary endpoint, PFS HR, OS HR, and ORR events and totals. For each randomized arm, we also recorded the original arm label, active treatment components, comparator type, biomarker-based eligibility, and the standardized treatment node used in the network meta-analysis. This arm-level mapping is presented in Table 1 to ensure that the classification of each intervention and comparator is traceable to the original trial report.

**Table 1:** Characteristics of included treatment comparisons and mapping of original trial arms to network nodes.

Study	Phase	Treatment 1	Treatment 2	N1/N2	Therapeutic category	Available outcomes	Original trial arm	Network node
Batchelor et al. (8)	III	Cediranib	Lomustine	131/65	Anti-angiogenic TKI vs. chemotherapy	PFS, OS, ORR	Cediranib vs. lomustine	Cediranib vs. Lomustine
	III	Cediranib + lomustine	Lomustine	129/65	Anti-angiogenic TKI + chemotherapy vs. chemotherapy	PFS, OS, ORR	Cediranib + lomustine vs. lomustine	Cediranib+Lomustine vs. Lomustine
Brandes et al. (34)	II	Galunisertib + lomustine	Lomustine	79/40	TGF-beta receptor inhibitor + chemotherapy vs. chemotherapy	OS, ORR	Galunisertib + lomustine vs. lomustine	Galunisertib+Lomustine vs. Lomustine
	II	Galunisertib	Lomustine	39/40	TGF-beta receptor inhibitor vs. chemotherapy	OS, ORR	Galunisertib vs. lomustine	Galunisertib vs. Lomustine
Brown et al. (35)	II	Cediranib + gefitinib	Cediranib	19/19	Dual targeted therapy vs. anti-angiogenic TKI	PFS, OS, ORR	Cediranib + gefitinib vs. cediranib	Cediranib+Gefitinib vs. Cediranib
Field et al. (36)	II	Bevacizumab + carboplatin	Bevacizumab	60/62	Anti-angiogenic + chemotherapy vs. anti-angiogenic monotherapy	PFS, OS, ORR	Bevacizumab + carboplatin vs. bevacizumab	Bevacizumab+Carboplatin vs. Bevacizumab
Tsien et al. (11)	II	Bevacizumab + re-irradiation	Bevacizumab	86/84	Anti-angiogenic + re-irradiation vs. anti-angiogenic monotherapy	PFS, OS	Bevacizumab + re-irradiation vs. bevacizumab	Bevacizumab+reRT vs. Bevacizumab
Stupp et al. (13)	III	TTF / NovoTTF-100A	Physician’s-choice active chemotherapy	120/117	Tumor-treating fields vs. chemotherapy	PFS, OS, ORR	NovoTTF-100A vs. physician’s-choice active chemotherapy	TTF vs. Lomustine*
Weathers et al. (37)	II	Bevacizumab + lomustine	Bevacizumab	35/36	Anti-angiogenic + chemotherapy vs. anti-angiogenic monotherapy	PFS, ORR	Bevacizumab + lomustine vs. bevacizumab	Bevacizumab+Lomustine vs. Bevacizumab
Wick et al. (38)	III	Enzastaurin	Lomustine	174/92	Targeted therapy vs. chemotherapy	PFS, OS, ORR	Enzastaurin vs. lomustine	Enzastaurin vs. Lomustine
Wick et al. (9)	III	Bevacizumab + lomustine	Lomustine	288/149	Anti-angiogenic + chemotherapy vs. chemotherapy	PFS, OS, ORR	Bevacizumab + lomustine vs. lomustine	Bevacizumab+Lomustine vs. Lomustine
Bota et al. (44)	II	ERC1671 + GM-CSF + cyclophosphamide + bevacizumab	Placebo + bevacizumab	4/4	Vaccine-based immunotherapy + anti-angiogenic vs. anti-angiogenic therapy	ORR	ERC1671 + GM-CSF + cyclophosphamide + bevacizumab vs. placebo + bevacizumab	ERC1671 combination+Bevacizumab vs. Bevacizumab
Reardon et al. (10)	III	Nivolumab	Bevacizumab	184/185	Immune checkpoint inhibitor vs. anti-angiogenic therapy	PFS, OS, ORR	Nivolumab vs. bevacizumab	Nivolumab vs. Bevacizumab
Brandes et al. (39)	II	Bevacizumab + lomustine	Lomustine	61/62	Anti-angiogenic + chemotherapy vs. chemotherapy	PFS, OS	Bevacizumab + lomustine vs. lomustine	Bevacizumab+Lomustine vs. Lomustine
Cloughesy et al. (40)	III	VB-111 + bevacizumab	Bevacizumab	128/128	Gene therapy + anti-angiogenic vs. anti-angiogenic monotherapy	PFS, OS, ORR	VB-111 + bevacizumab vs. bevacizumab	VB-111 + Bevacizumab vs. Bevacizumab
Reardon et al. (12)	II	Rindopepimut + bevacizumab	Bevacizumab	36/37	Peptide vaccine + anti-angiogenic vs. anti-angiogenic therapy	PFS, OS, ORR	Rindopepimut + bevacizumab vs. bevacizumab (EGFRvIII-positive population)	Rindopepimut+Bevacizumab vs. Bevacizumab
Puduvalli et al. (41)	II	Bevacizumab + vorinostat	Bevacizumab	49/41	Anti-angiogenic + HDAC inhibitor vs. anti-angiogenic monotherapy	PFS, OS	Bevacizumab + vorinostat vs. bevacizumab	Bevacizumab+Vorinostat vs. Bevacizumab
Galanis et al. (42)	II	Bevacizumab + dasatinib	Bevacizumab	83/38	Anti-angiogenic + Src inhibitor vs. anti-angiogenic monotherapy	PFS, OS, ORR	Bevacizumab + dasatinib vs. bevacizumab	Bevacizumab+Dasatinib vs. Bevacizumab
Lee et al. (43)	II	Bevacizumab + trebananib	Bevacizumab	57/58	Anti-angiogenic + angiopoietin inhibitor vs. anti-angiogenic monotherapy	PFS, OS, ORR	Bevacizumab + trebananib vs. bevacizumab	Bevacizumab+Trebananib vs. Bevacizumab
Wen et al. (14)	II/III	Regorafenib	Lomustine	127/128	Multikinase inhibitor vs. chemotherapy	PFS, OS	Regorafenib vs. platform control (lomustine in the recurrent-disease cohort)	Regorafenib vs. Lomustine
Lombardi et al. (45)	II	Regorafenib	Lomustine	59/60	Multikinase inhibitor vs. chemotherapy	PFS, OS, ORR	Regorafenib vs. lomustine	Regorafenib vs. Lomustine

EGFRvIII, epidermal growth factor receptor variant III; GM-CSF, granulocyte-macrophage colony-stimulating factor; HDAC, histone deacetylase; ORR, objective response rate; OS, overall survival; PFS, progression-free survival; TGF, transforming growth factor; TKI, tyrosine kinase inhibitor; TTF, tumor-treating fields. Original trial arm records the randomized arm labels reported by each study; Network node records the standardized node names used in the current network meta-analysis. Combination regimens were retained as separate nodes, and biomarker-defined eligibility was recorded independently of node identity. *Stupp 2012 compared NovoTTF-100A with physician’s-choice active chemotherapy rather than lomustine alone. In the present analysis, this heterogeneous control arm was mapped to the lomustine node to maintain network connectivity. Because the comparator was not restricted to lomustine alone, this mapping may have introduced additional clinical heterogeneity; estimates involving tumor-treating fields should therefore be interpreted cautiously.

When available, molecular and biomarker-related variables, including MGMT promoter methylation status, IDH mutation status, and EGFRvIII expression, were also collected because these factors may influence prognosis, treatment eligibility, and interpretation of therapeutic effects in recurrent glioblastoma. For trials that included both newly diagnosed and recurrent glioblastoma cohorts, only data from the recurrent-disease cohort were extracted when available.

When HRs were not directly reported, they were estimated from available published data or reconstructed from published Kaplan–Meier curves when feasible, using established methods (26, 27). For ORR, the number of patients achieving complete or partial response and the total number of evaluable patients in each treatment arm were extracted to calculate ORs. Disagreements between reviewers were resolved by discussion or by consultation with a third reviewer.

### 2.3 Risk of bias assessment

Risk of bias in the included randomized trials was assessed using the Cochrane risk-of-bias tool for randomized trials, which evaluates random sequence generation, allocation concealment, blinding of participants and personnel, blinding of outcome assessment, incomplete outcome data, selective reporting, and other potential sources of bias (28). Each domain was judged as having a low, high, or unclear risk of bias. Although the revised RoB 2 tool has been developed for assessing bias in randomized trials, the domain-based Cochrane tool was used in the present review because it corresponded to the reporting structure available in the included trials and allowed consistent assessment across trials conducted over different periods (29). Recent methodological studies have also noted that RoB 2 may provide a more detailed assessment but can be challenging and resource-intensive in practice (30, 31).

Two reviewers independently performed the risk-of-bias assessment. Any discrepancies were first resolved through discussion, and unresolved disagreements were adjudicated by consultation with a third reviewer. The risk-of-bias assessment was used to contextualize the reliability and interpretability of treatment effects and rankings, but no study was excluded solely on the basis of risk-of-bias judgments.

### 2.4 Statistical analysis

A frequentist network meta-analysis was performed separately for each efficacy outcome. HRs with 95% CIs were used for PFS and OS, whereas ORs with 95% CIs were used for ORR. Random-effects models were selected *a priori* because substantial clinical and methodological heterogeneity was anticipated across trials. A common-effect model was not considered clinically appropriate because it assumes a single underlying treatment effect across heterogeneous trial populations and treatment settings. Prediction intervals were not presented because the networks were sparse and many direct comparisons were informed by a single trial, resulting in limited information for reliable estimation of the expected distribution of treatment effects. Analyses were performed using R software, primarily with the netmeta package (32).

Treatment nodes were defined primarily according to the active treatment components actually received in each randomized arm. Monotherapies containing the same active agent were assigned to the same node when there were no clinically meaningful differences in dose, schedule, route of administration, or overall treatment strategy. Combination regimens were retained as separate nodes from their component monotherapies; for example, bevacizumab, lomustine, and bevacizumab plus lomustine were represented as distinct nodes. Regimens were not combined solely because they belonged to the same broad therapeutic class.

Original trial-arm labels and their corresponding standardized network nodes are reported in Table 1. Biomarker-defined eligibility was treated as a population characteristic rather than as a separate pharmacological node. For example, rindopepimut plus bevacizumab was classified according to its active treatment components, while the restriction to patients with EGFRvIII-positive tumors was recorded separately and considered in the assessment of transitivity and generalizability. Similarly, placebo-containing control regimens were classified according to the active treatment received; therefore, placebo plus bevacizumab was mapped to the bevacizumab node.

Multi-arm and platform trials were retained under their original study identity so that comparisons sharing a common control group were not treated as independent studies. For trials using complex control descriptions, including physician’s-choice chemotherapy or platform-trial controls, both the original trial-arm label and the node used in the analysis are explicitly reported in Table 1. This approach allows readers to evaluate the clinical appropriateness of each node assignment and improves the reproducibility of the network structure.

The transitivity assumption was assessed clinically by examining the distribution of potential treatment-effect modifiers across the included trials and direct comparisons. The factors considered included recurrence line, prior exposure to bevacizumab or lomustine, baseline performance status, response-assessment criteria, MGMT promoter methylation status, IDH mutation status, EGFRvIII expression, biomarker-based eligibility, and treatment context. Formal subgroup analyses and network meta-regression were not performed because these variables were incompletely and inconsistently reported, subgroup-specific comparative estimates were rarely available, and several potential effect modifiers were closely associated with individual treatment nodes.

For graphical presentation, treatment networks were constructed with nodes representing interventions and edges representing direct comparisons. Node size reflected the number of patients assigned to each treatment, whereas line thickness reflected the amount of direct comparative evidence.

Treatment rankings were summarized using P-scores. Higher P-scores indicated a more favorable relative position within the network. However, P-scores were treated as descriptive and exploratory measures and were not interpreted as evidence of statistical significance or clinically proven superiority. P-score figures were presented as exploratory visual summaries and were interpreted only in conjunction with the corresponding forest plots, effect estimates, 95% CIs, sample sizes, and amount of direct evidence. Accordingly, treatment rankings were interpreted only after consideration of the corresponding effect estimates, 95% CIs, sample sizes, and precision of the underlying evidence (21). Global inconsistency was assessed separately for the PFS, OS, and ORR networks. Given the sparse network structure and the limited number of closed loops, local node-splitting analyses were not presented. The results of the global inconsistency assessment are reported in Supplementary Table S1. Because several comparisons were supported by limited direct evidence, small-study effects and publication bias were not formally assessed using comparison-adjusted funnel plots (33).

## 3 Results

### 3.1 Study selection

**Figure 1:**
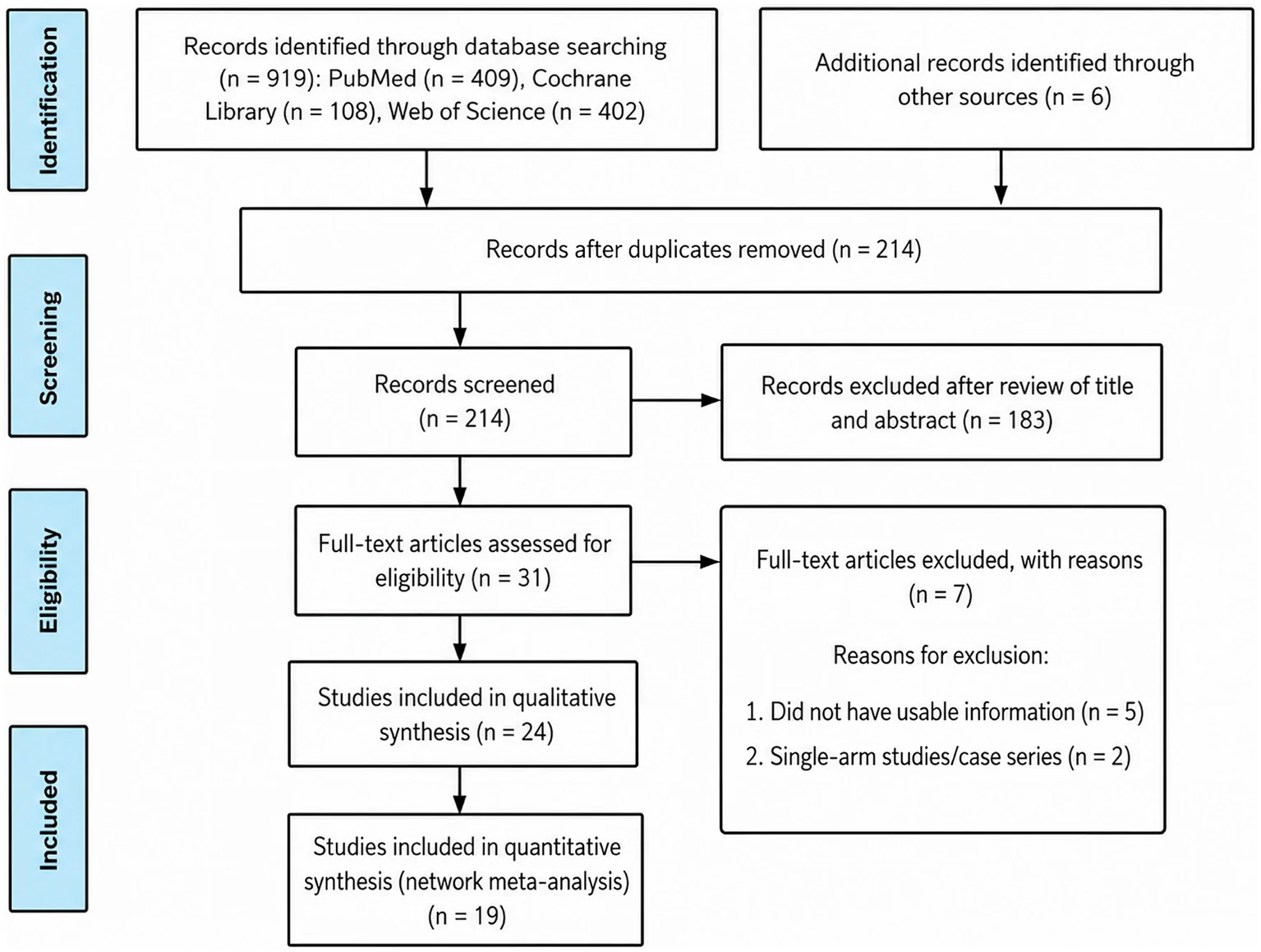
PRISMA flow diagram of study selection. The flow diagram shows the process of study identification, screening, eligibility assessment, and inclusion. A total of 919 records were identified through database searching, and 6 additional records were identified through other sources. After duplicate removal and screening, 24 studies were included in the qualitative synthesis, of which 19 contributed to the quantitative network meta-analysis.

The study selection process is shown in Figure 1. A total of 919 records were identified through database searching, including 409 from PubMed, 108 from the Cochrane Library, and 402 from Web of Science. Six additional records were identified through manual screening of reference lists and other relevant sources. After duplicate removal, 214 records remained for title and abstract screening. Of these, 183 records were excluded because they did not meet the prespecified eligibility criteria, including non-randomized studies, non-recurrent disease settings, non-glioblastoma populations, reviews, protocols, preclinical studies, or reports without relevant efficacy outcomes.

Thirty-one full-text articles were subsequently assessed for eligibility. Seven articles were excluded after full-text review, including five studies with insufficient usable data for effect-size estimation and two studies with single-arm or case-series design. Finally, 24 studies were included in the qualitative synthesis. Of these, 19 studies (8–14, 34–45) contributed data to at least one quantitative network meta-analysis for PFS, OS, or ORR, whereas five studies were not synthesized quantitatively because they lacked analyzable data for the prespecified outcomes or were not connected to the main treatment network.

### 3.2 Characteristics of included studies

The included trials covered several major therapeutic approaches used or tested in recurrent glioblastoma, including anti-angiogenic therapy, alkylating chemotherapy, multi-target tyrosine kinase inhibitors, immune checkpoint inhibition, vaccine-based immunotherapy, tumor-treating fields, gene therapy combined with bevacizumab, and re-irradiation combined with bevacizumab (8–14, 34–45). Bevacizumab- and lomustine-containing regimens served as the main comparator or backbone regimens in the evidence network, as summarized in Table 1. Most trials primarily enrolled patients at first recurrence, although several studies also included patients receiving second-line or later therapies, contributing to heterogeneity in prior treatment exposure and disease course. Molecular characteristics, including MGMT promoter methylation status, IDH mutation status, and EGFRvIII expression, were incompletely reported across studies. This limited the ability to perform biomarker-stratified analyses and may affect interpretation of treatment rankings, particularly for biomarker-defined regimens such as rindopepimut plus bevacizumab, which was evaluated in EGFRvIII-positive recurrent glioblastoma (12). The incomplete molecular characterization of older trials is also relevant because contemporary glioblastoma classification and clinical interpretation increasingly rely on integrated molecular diagnosis, particularly IDH status (1, 2). Although 19 studies contributed to the quantitative network meta-analysis, some multi-arm trials contributed more than one treatment comparison; therefore, Table 1 summarizes the treatment comparisons included in the evidence network. To improve transparency regarding treatment-node construction, Table 1 additionally presents the original randomized arm labels and the corresponding standardized network nodes used in the analyses. Most treatment arms were mapped directly according to their named active components. Combination regimens were retained as distinct nodes, placebo-containing regimens were classified according to the active treatment received, and biomarker-defined eligibility was recorded separately from treatment-node identity. For multi-arm, physician’s-choice, and platform-trial comparisons, the original arm description was retained alongside the assigned network node to make the classification process explicit.

A structured clinical assessment of the transitivity assumption was conducted using the trial-level characteristics available in the included studies. At the general disease-setting level, the trials were broadly comparable because they predominantly enrolled adults with recurrent or refractory glioblastoma after previous standard treatment. Most studies primarily included patients at first recurrence and required a relatively preserved baseline performance status, supporting the overall clinical plausibility of indirect comparisons within the network.

However, several potential treatment-effect modifiers differed across trials or were incompletely reported. Some studies included patients receiving treatment at second or later recurrence, and prior exposure to bevacizumab or lomustine was not consistently reported or permitted. Performance-status eligibility thresholds varied, and radiographic outcomes were assessed mainly using either the Macdonald or RANO criteria. Molecular comparability was also limited because MGMT promoter methylation and IDH mutation status were frequently unreported in older trials, whereas rindopepimut-based therapy was evaluated specifically in an EGFRvIII-positive population. Therefore, the transitivity assumption was considered broadly plausible at the overall recurrent-glioblastoma disease-setting level but could not be fully verified for every indirect comparison. Comparisons involving biomarker-selected populations, later-line treatment settings, and radiographic response outcomes should consequently be interpreted with caution.

### 3.3 Network structure and consistency assessment

**Figure 2:**
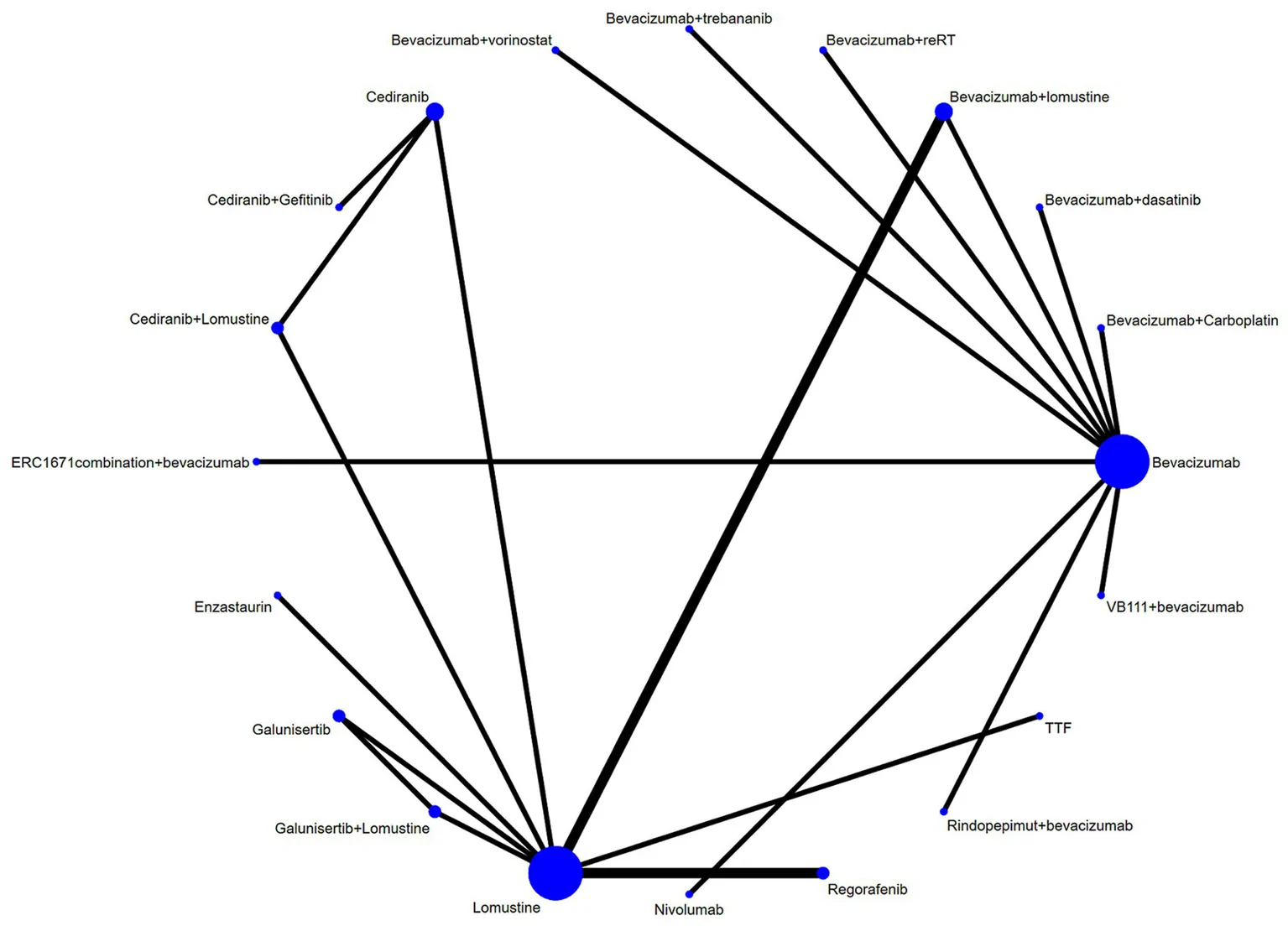
Treatment network of eligible comparisons included in the network meta-analysis. Each node represents a treatment strategy, and each edge represents a direct comparison between two treatments. Node size is proportional to the number of patients assigned to each treatment, and line thickness is proportional to the amount of direct comparative evidence. ERC1671-based regimen refers to ERC1671 + GM-CSF + cyclophosphamide + bevacizumab. GM-CSF, granulocyte-macrophage colony-stimulating factor; reRT, re-irradiation; TTF, tumor-treating fields; VB-111, ofranergene obadenovec.

The treatment network is shown in Figure 2. Bevacizumab and lomustine constituted the two major comparator nodes in the evidence network. Bevacizumab monotherapy was directly connected with nivolumab and multiple bevacizumab-based combination regimens, including rindopepimut plus bevacizumab, bevacizumab plus re-irradiation, bevacizumab plus dasatinib, bevacizumab plus carboplatin, bevacizumab plus vorinostat, bevacizumab plus trebananib, VB-111 plus bevacizumab, and the ERC1671-based regimen (10–12, 36, 37, 40–44). Lomustine served as another central comparator and was directly connected with cediranib, cediranib plus lomustine, galunisertib-based regimens, enzastaurin, regorafenib, tumor-treating fields, and bevacizumab plus lomustine (8, 9, 13, 14, 34, 38, 39, 45). The central roles of bevacizumab and lomustine enabled indirect comparisons across a broad range of therapeutic strategies, but they also indicate that many relative treatment effects were dependent on these central comparators rather than on extensive direct head-to-head evidence between all active regimens.

Global inconsistency testing did not identify statistically significant inconsistency for PFS, OS, or ORR, with *p* values of 0.3127, 0.3482, and 0.8735, respectively. The global inconsistency p values are provided in Supplementary Table S1. However, because the evidence networks were sparse and several treatment comparisons were supported by limited direct evidence, these tests may have had insufficient power to detect moderate inconsistency. Furthermore, the network structure depended heavily on bevacizumab and lomustine as central comparator nodes, and many indirect estimates were therefore derived through these treatments rather than supported by multiple independent closed loops. Therefore, the non-significant global inconsistency tests should be interpreted as indicating that no statistically significant inconsistency was detected, rather than as definitive confirmation of network consistency.

### 3.4 Risk of bias assessment

**Figure 3:**
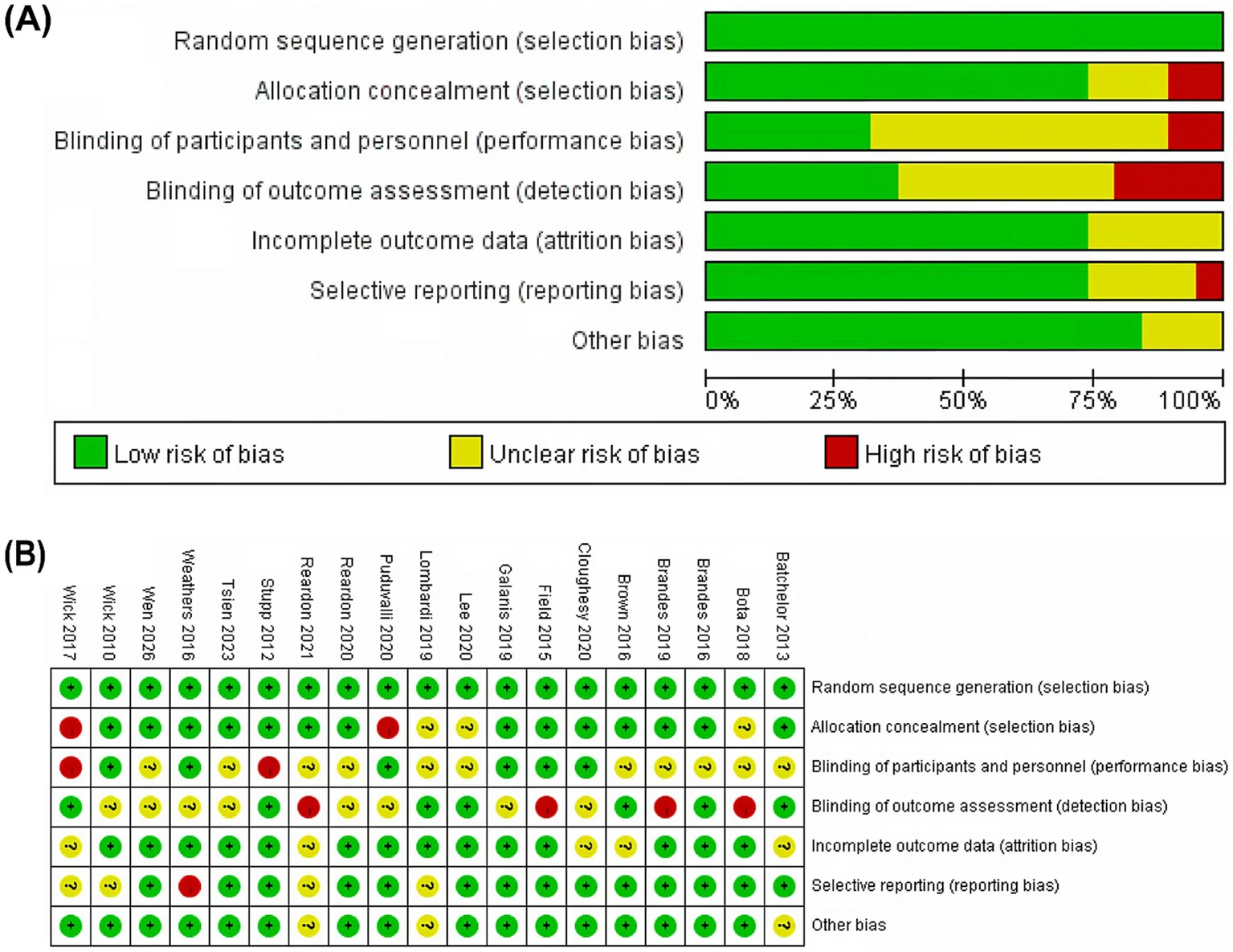
Risk of bias assessment of included studies. **(A)** Overall risk-of-bias graph. **(B)** Study-level risk-of-bias summary. Green indicates low risk of bias, yellow indicates unclear risk of bias, and red indicates high risk of bias.

The overall and study-level risk-of-bias assessments are shown in Figure 3. Most included studies were judged to have a low risk of bias for random sequence generation, incomplete outcome data, and selective reporting. However, allocation concealment and blinding procedures were insufficiently reported in several trials. In addition, open-label designs contributed to unclear or high risk in the domains of performance and detection bias.

The potential implications of these judgments differed across outcomes. Open-label designs, incomplete blinding of outcome assessors, and variation in radiographic assessment procedures may have had a greater influence on PFS and ORR than on OS. Because PFS and ORR depend on imaging interpretation and the timing of disease assessment, these outcomes may be particularly susceptible to detection bias, especially in trials of bevacizumab-containing regimens. By contrast, OS is a more objective endpoint, although it may still be influenced by crossover and subsequent therapies. The assessment was based on the domain-based Cochrane risk-of-bias tool for randomized trials (28).

### 3.5 Progression-free survival

**Figure 4:**
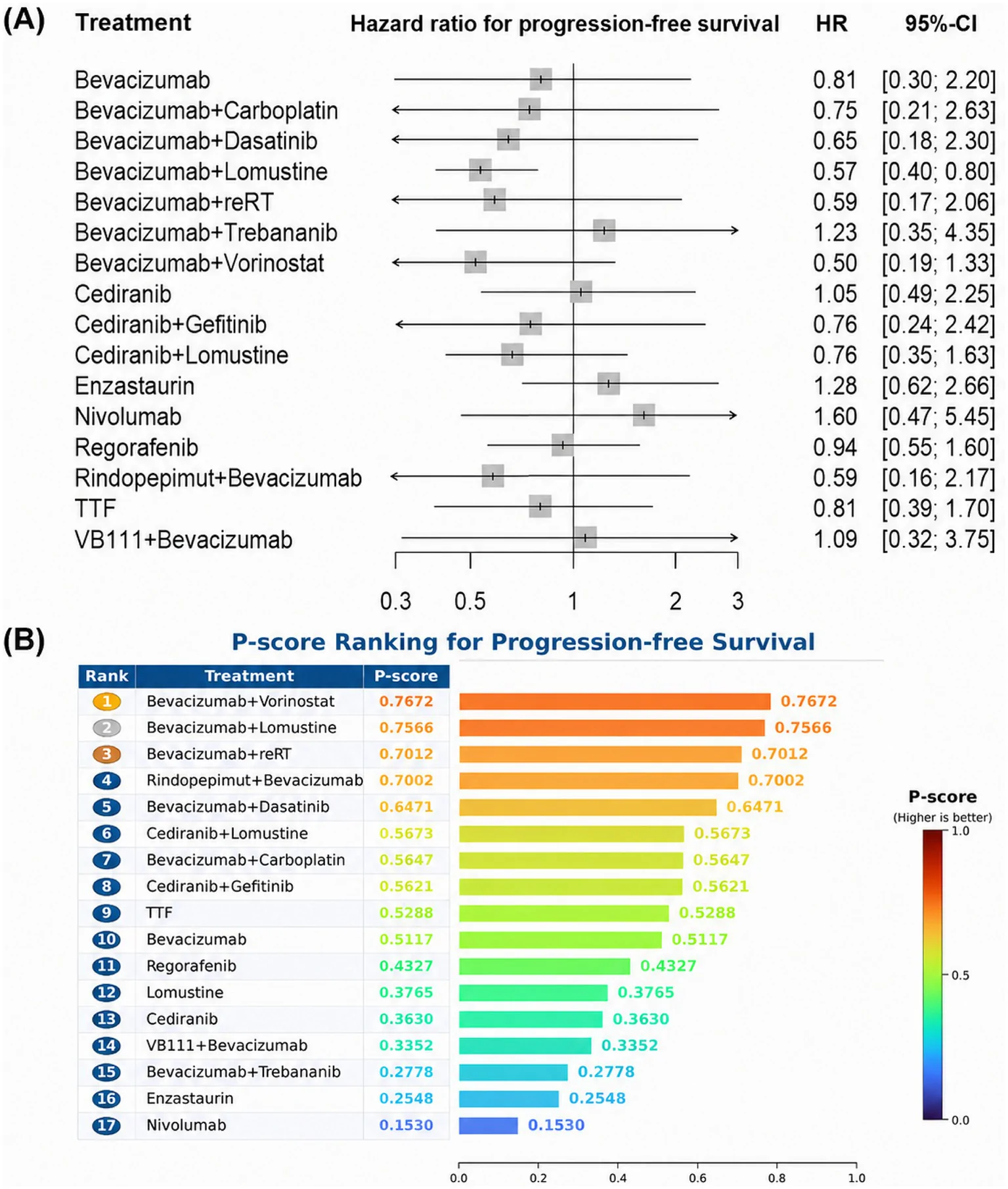
Network meta-analysis results for progression-free survival. **(A)** Forest plot comparing therapeutic strategies with lomustine as the reference treatment. HR < 1 favors the treatment listed on the left, whereas HR > 1 favors lomustine. **(B)** Exploratory P-score ranking for progression-free survival. Higher P-scores indicate more favorable numerical rankings within the network but should not be interpreted as evidence of statistical significance or clinically proven superiority. CI, confidence interval; HR, hazard ratio; PFS, progression-free survival.

For the PFS analysis, lomustine was used as the reference treatment. The PFS forest plot and P-score ranking are shown in Figures 4A,B, respectively. Compared with lomustine, bevacizumab plus lomustine was associated with a statistically significant reduction in the risk of disease progression or death (HR = 0.57, 95% CI: 0.40–0.80). Bevacizumab plus vorinostat, bevacizumab plus re-irradiation, rindopepimut plus bevacizumab, bevacizumab plus dasatinib, bevacizumab plus carboplatin, cediranib plus gefitinib, cediranib plus lomustine, tumor-treating fields, and bevacizumab monotherapy all had HRs below 1.0, but their 95% CIs crossed 1.0.

Among the evaluated treatments, bevacizumab plus vorinostat had the highest numerical P-score (P-score = 0.7672), followed by bevacizumab plus lomustine (P-score = 0.7566), bevacizumab plus re-irradiation (P-score = 0.7012), rindopepimut plus bevacizumab (P-score = 0.7002), and bevacizumab plus dasatinib (P-score = 0.6471). However, the estimated effect of bevacizumab plus vorinostat was not statistically significant compared with lomustine (HR = 0.50, 95% CI: 0.19–1.33). In contrast, bevacizumab plus lomustine had the second-highest numerical P-score and was the only regimen associated with a statistically significant PFS benefit compared with lomustine. Therefore, the P-score ranking should be regarded as descriptive rather than as evidence that bevacizumab plus vorinostat was superior.

To evaluate the robustness of the findings, a sensitivity analysis excluding trials with fewer than 20 patients per arm was performed. The corresponding forest plot and P-score ranking are shown in Supplementary Figures 1a,b, respectively. Compared with lomustine, bevacizumab plus lomustine remained associated with a statistically significant reduction in the risk of disease progression or death (HR = 0.58, 95% CI: 0.34–0.97). Bevacizumab plus vorinostat (HR = 0.51, 95% CI: 0.14–1.89), bevacizumab plus re-irradiation (HR = 0.59, 95% CI: 0.17–2.06), rindopepimut plus bevacizumab (HR = 0.59, 95% CI: 0.16–2.17), bevacizumab plus dasatinib (HR = 0.65, 95% CI: 0.18–2.30), bevacizumab plus carboplatin (HR = 0.75, 95% CI: 0.21–2.63), cediranib plus lomustine (HR = 0.76, 95% CI: 0.35–1.63), tumor-treating fields (HR = 0.81, 95% CI: 0.39–1.70), bevacizumab monotherapy (HR = 0.81, 95% CI: 0.30–2.20), and regorafenib (HR = 0.94, 95% CI: 0.55–1.60) all had HRs below 1.0, but their 95% CIs crossed 1.0. In contrast, bevacizumab plus trebananib, cediranib, enzastaurin, nivolumab, and VB-111 plus bevacizumab had HRs above 1.0, with 95% CIs also crossing 1.0.

In the sensitivity analysis, bevacizumab plus vorinostat again had the highest numerical P-score (P-score = 0.7738), followed by bevacizumab plus lomustine (P-score = 0.7631), bevacizumab plus re-irradiation (P-score = 0.7073), rindopepimut plus bevacizumab (P-score = 0.7061), and bevacizumab plus dasatinib (P-score = 0.6524). Nevertheless, the effect estimate for bevacizumab plus vorinostat remained statistically non-significant (HR = 0.51, 95% CI: 0.14–1.89), whereas bevacizumab plus lomustine remained significantly associated with improved PFS. The relative numerical rankings were largely unchanged, but they should not be interpreted as evidence of comparative superiority. Cediranib plus gefitinib was no longer included because the corresponding Brown 2016 trial was excluded.

### 3.6 Overall survival

**Figure 5:**
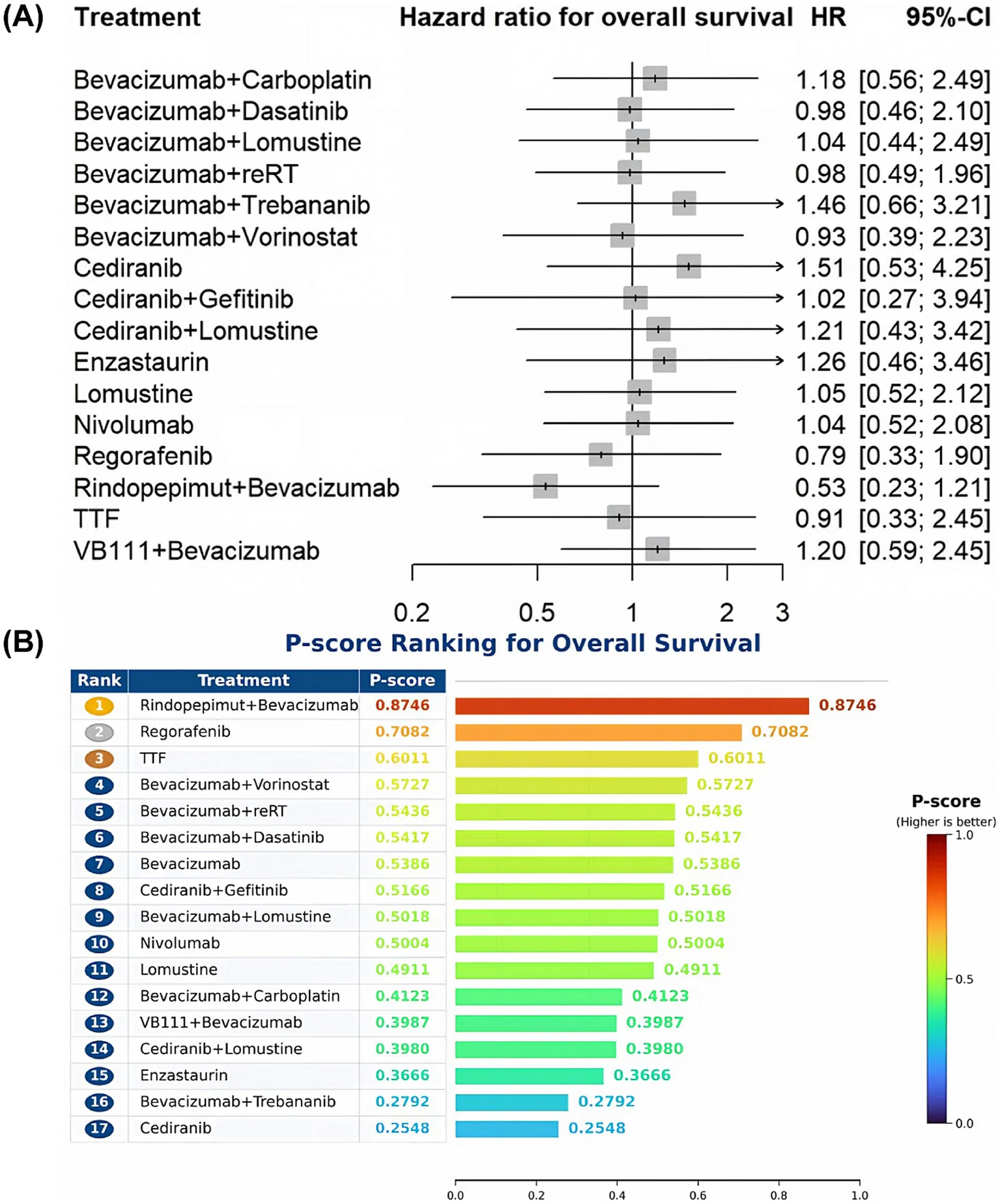
Network meta-analysis results for overall survival. **(A)** Forest plot comparing therapeutic strategies with bevacizumab as the reference treatment. HR < 1 favors the treatment listed on the left, whereas HR > 1 favors bevacizumab. **(B)** Exploratory P-score ranking for overall survival. Higher P-scores indicate more favorable numerical rankings within the network but should not be interpreted as evidence of statistical significance or clinically proven survival superiority. CI, confidence interval; HR, hazard ratio; OS, overall survival.

For the OS analysis, bevacizumab was used as the reference treatment because it was a central comparator in the evidence network; this analytical choice should not be interpreted as evidence of established survival superiority. The OS forest plot and P-score ranking are shown in Figures 5A,B, respectively. None of the evaluated treatments demonstrated a statistically significant difference in the risk of death compared with bevacizumab, as all 95% CIs crossed 1.0.

Rindopepimut plus bevacizumab showed the largest numerical reduction in the risk of death (HR = 0.53, 95% CI: 0.23–1.21), followed by regorafenib (HR = 0.79, 95% CI: 0.33–1.90), tumor-treating fields (HR = 0.91, 95% CI: 0.33–2.45), bevacizumab plus vorinostat (HR = 0.93, 95% CI: 0.39–2.23), bevacizumab plus dasatinib (HR = 0.98, 95% CI: 0.46–2.10), and bevacizumab plus re-irradiation (HR = 0.98, 95% CI: 0.49–1.96). Although these treatments had HRs below 1.0, their 95% CIs crossed 1.0.

In contrast, cediranib (HR = 1.51, 95% CI: 0.53–4.25), bevacizumab plus trebananib (HR = 1.46, 95% CI: 0.66–3.21), enzastaurin (HR = 1.26, 95% CI: 0.46–3.46), cediranib plus lomustine (HR = 1.21, 95% CI: 0.43–3.42), VB-111 plus bevacizumab (HR = 1.20, 95% CI: 0.59–2.45), bevacizumab plus carboplatin (HR = 1.18, 95% CI: 0.56–2.49), lomustine (HR = 1.05, 95% CI: 0.52–2.12), nivolumab (HR = 1.04, 95% CI: 0.52–2.08), bevacizumab plus lomustine (HR = 1.04, 95% CI: 0.44–2.49), and cediranib plus gefitinib (HR = 1.02, 95% CI: 0.27–3.94) had HRs above 1.0, but none of these differences reached statistical significance.

Rindopepimut plus bevacizumab had the highest numerical P-score for OS (P-score = 0.8746), followed by regorafenib (P-score = 0.7082), tumor-treating fields (P-score = 0.6011), bevacizumab plus vorinostat (P-score = 0.5727), and bevacizumab plus re-irradiation (P-score = 0.5436). However, none of these treatments demonstrated a statistically significant OS benefit compared with bevacizumab, as all corresponding 95% CIs crossed 1.0. These P-score results therefore represent exploratory relative rankings rather than evidence of proven survival superiority.

To assess the robustness of the findings, a sensitivity analysis excluding trials with fewer than 20 patients per arm was performed. The corresponding forest plot and P-score ranking are shown in Supplementary Figures 2a,b, respectively. No statistically significant differences in OS were observed between any retained treatment and bevacizumab.

Rindopepimut plus bevacizumab remained associated with the largest numerical reduction in the risk of death (HR = 0.53, 95% CI: 0.23–1.21), followed by regorafenib (HR = 0.79, 95% CI: 0.33–1.90), tumor-treating fields (HR = 0.91, 95% CI: 0.33–2.45), bevacizumab plus vorinostat (HR = 0.93, 95% CI: 0.39–2.23), bevacizumab plus dasatinib (HR = 0.98, 95% CI: 0.46–2.10), and bevacizumab plus re-irradiation (HR = 0.98, 95% CI: 0.49–1.96). However, the 95% CIs for all these estimates crossed 1.0.

In the sensitivity analysis, rindopepimut plus bevacizumab retained the highest numerical P-score (P-score = 0.8801), followed by regorafenib (P-score = 0.7119), tumor-treating fields (P-score = 0.6031), bevacizumab plus vorinostat (P-score = 0.5744), and bevacizumab plus re-irradiation (P-score = 0.5450). Although the relative ordering of these treatments remained unchanged, none demonstrated a statistically significant OS advantage over bevacizumab. The stability of the numerical rankings should therefore not be interpreted as confirmation of comparative efficacy. Cediranib plus gefitinib was no longer included because the corresponding Brown 2016 trial was excluded.

### 3.7 Objective response rate

**Figure 6:**
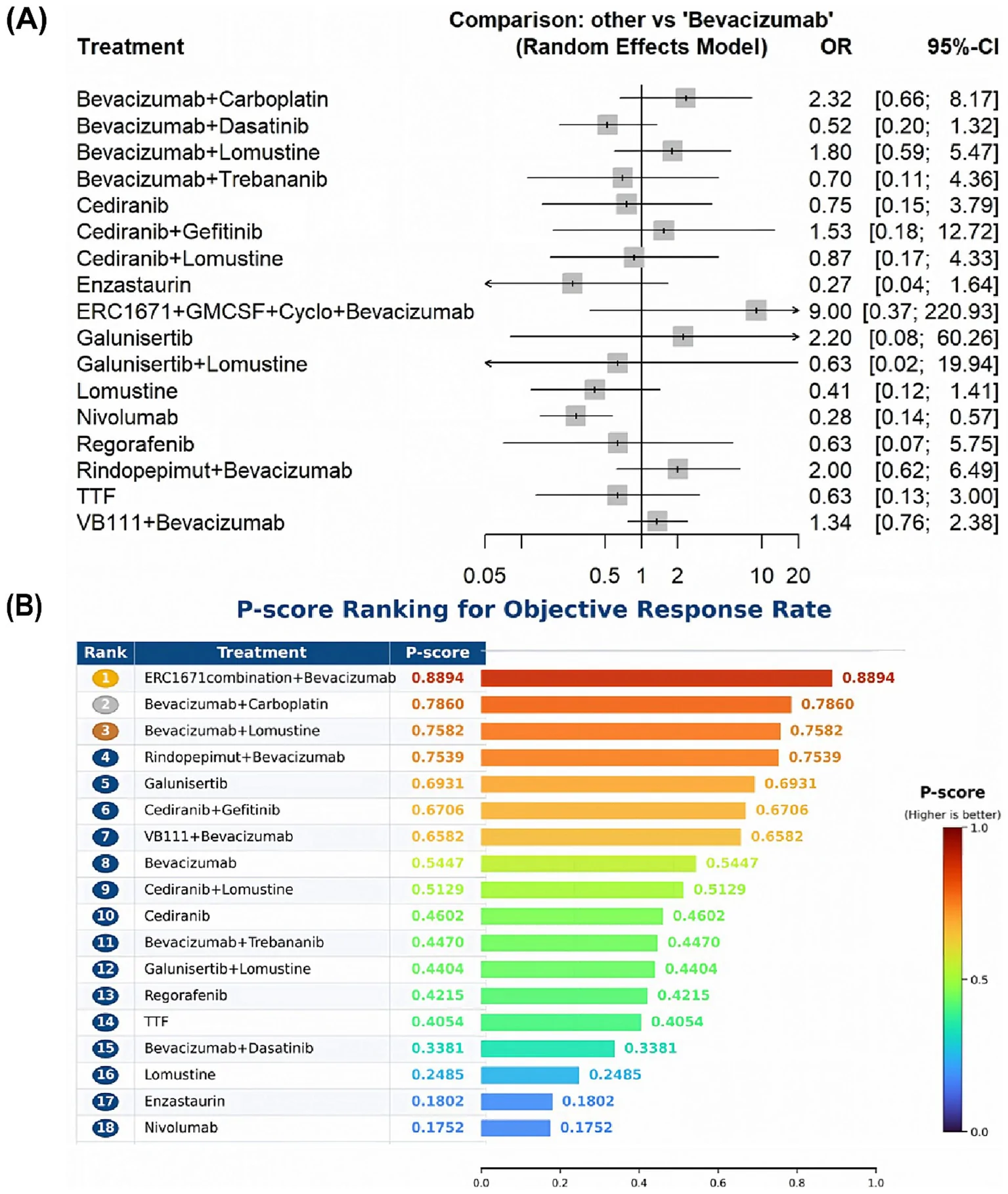
Network meta-analysis results for objective response rate. **(A)** Forest plot comparing therapeutic strategies with bevacizumab as the reference treatment. *OR* > 1 favors the treatment listed on the left, whereas *OR* < 1 favors bevacizumab. **(B)** Exploratory P-score ranking for objective response rate. Higher P-scores indicate more favorable numerical rankings within the network but should not be interpreted as evidence of statistical significance or clinically proven superiority. *CI*, confidence interval; *OR*, odds ratio; *ORR*, objective response rate.

For the ORR analysis, bevacizumab was used as the reference treatment. The ORR forest plot and P-score ranking are shown in Figures 6A,B, respectively. Compared with bevacizumab, none of the evaluated treatments showed a statistically significant improvement in objective response rate. In contrast, nivolumab was associated with significantly lower odds of objective response than bevacizumab (OR = 0.28, 95% CI: 0.14–0.57).

Bevacizumab plus carboplatin (OR = 2.32, 95% CI: 0.66–8.17), bevacizumab plus lomustine (OR = 1.80, 95% CI: 0.59–5.47), cediranib plus gefitinib (OR = 1.53, 95% CI: 0.18–12.72), ERC1671 combination plus bevacizumab (OR = 9.00, 95% CI: 0.37–220.93), galunisertib (OR = 2.20, 95% CI: 0.08–60.26), rindopepimut plus bevacizumab (OR = 2.00, 95% CI: 0.62–6.49), and VB-111 plus bevacizumab (OR = 1.34, 95% CI: 0.76–2.38) had ORs above 1.0, but their 95% CIs crossed 1.0. Bevacizumab plus dasatinib (OR = 0.52, 95% CI: 0.20–1.32), bevacizumab plus trebananib (OR = 0.70, 95% CI: 0.11–4.36), cediranib (OR = 0.75, 95% CI: 0.15–3.79), cediranib plus lomustine (OR = 0.87, 95% CI: 0.17–4.33), enzastaurin (OR = 0.27, 95% CI: 0.04–1.64), galunisertib plus lomustine (OR = 0.63, 95% CI: 0.02–19.94), lomustine (OR = 0.41, 95% CI: 0.12–1.41), regorafenib (OR = 0.63, 95% CI: 0.07–5.75), and tumor-treating fields (OR = 0.63, 95% CI: 0.13–3.00) had ORs below 1.0, but their 95% CIs also crossed 1.0.

ERC1671 combination plus bevacizumab had the highest numerical P-score for ORR (P-score = 0.8894), followed by bevacizumab plus carboplatin (P-score = 0.7860), bevacizumab plus lomustine (P-score = 0.7582), rindopepimut plus bevacizumab (P-score = 0.7539), and galunisertib (P-score = 0.6931). However, the effect estimate for the ERC1671-based regimen was highly imprecise and not statistically significant (OR = 9.00, 95% CI: 0.37–220.93), and the supporting trial included only four patients per treatment arm. Therefore, its high P-score should not be interpreted as evidence of established efficacy or superiority.

To evaluate the robustness of the findings, a sensitivity analysis excluding trials with fewer than 20 patients per arm was performed. The corresponding forest plot and P-score ranking are shown in Supplementary Figures 3a,b, respectively. In the sensitivity analysis, no retained treatment demonstrated a statistically significant improvement in objective response rate compared with bevacizumab, whereas nivolumab remained associated with significantly lower odds of objective response (OR = 0.28, 95% CI: 0.14–0.57).

Bevacizumab plus carboplatin (OR = 2.32, 95% CI: 0.66–8.17), bevacizumab plus lomustine (OR = 1.80, 95% CI: 0.59–5.47), galunisertib (OR = 2.20, 95% CI: 0.08–60.26), rindopepimut plus bevacizumab (OR = 2.00, 95% CI: 0.62–6.49), and VB-111 plus bevacizumab (OR = 1.34, 95% CI: 0.76–2.38) all had ORs above 1.0, but their 95% CIs crossed 1.0. Similarly, bevacizumab plus dasatinib (OR = 0.52, 95% CI: 0.20–1.32), bevacizumab plus trebananib (OR = 0.70, 95% CI: 0.11–4.36), cediranib (OR = 0.75, 95% CI: 0.15–3.79), cediranib plus lomustine (OR = 0.87, 95% CI: 0.17–4.33), enzastaurin (OR = 0.27, 95% CI: 0.04–1.64), galunisertib plus lomustine (OR = 0.63, 95% CI: 0.02–19.94), lomustine (OR = 0.41, 95% CI: 0.12–1.41), regorafenib (OR = 0.63, 95% CI: 0.07–5.75), and tumor-treating fields (OR = 0.63, 95% CI: 0.13–3.00) had ORs below 1.0, with 95% CIs also crossing 1.0.

After exclusion of trials with fewer than 20 patients per arm, bevacizumab plus carboplatin had the highest numerical P-score (P-score = 0.8222), followed by bevacizumab plus lomustine (P-score = 0.7964), rindopepimut plus bevacizumab (P-score = 0.7888), galunisertib (P-score = 0.7137), and VB-111 plus bevacizumab (P-score = 0.6882). However, none of these regimens demonstrated a statistically significant improvement in ORR compared with bevacizumab. Thus, these P-scores represent descriptive relative rankings only. The ERC1671 combination plus bevacizumab and cediranib plus gefitinib were no longer included after exclusion of the corresponding small trials.

## 4 Discussion

This frequentist network meta-analysis compared multiple therapeutic strategies for recurrent or refractory glioblastoma across progression-free survival, overall survival, and objective response rate. The updated analysis incorporated the REGOMA trial and included sensitivity analyses excluding trials with fewer than 20 patients in at least one treatment arm. Three principal findings emerged. First, bevacizumab plus lomustine was the only treatment associated with a statistically significant improvement in PFS compared with lomustine. Second, no evaluated treatment produced a statistically significant OS advantage over bevacizumab. Although rindopepimut plus bevacizumab and regorafenib had favorable numerical OS P-scores, these rankings should not be regarded as sufficient grounds for routine treatment selection. Third, no treatment significantly improved ORR relative to bevacizumab, whereas nivolumab was associated with significantly lower odds of objective response. The sensitivity analyses generally supported the stability of the PFS and OS findings but also demonstrated that the ORR rankings were more vulnerable to the exclusion of small studies.

For PFS, bevacizumab plus lomustine significantly reduced the risk of progression or death compared with lomustine (9). This finding remained statistically significant after trials with fewer than 20 patients per arm were excluded, suggesting that the observed PFS benefit was not driven solely by small studies. Several other regimens, including bevacizumab plus vorinostat, bevacizumab plus re-irradiation, rindopepimut plus bevacizumab, and bevacizumab plus dasatinib, produced HRs below 1.0 but had wide confidence intervals that crossed the null value (11, 12, 41, 42). Therefore, although these treatments achieved relatively favorable P-score rankings, the available evidence does not establish a definitive PFS benefit.

Bevacizumab plus vorinostat ranked first for PFS, followed closely by bevacizumab plus lomustine. This apparent discrepancy between the effect estimates and the ranking results illustrates an important limitation of treatment rankings. P-scores reflect the relative position of each intervention within the network and are influenced by both the magnitude and precision of the estimated effects (21). A high P-score does not necessarily indicate a statistically significant or clinically established advantage. In the present analysis, bevacizumab plus vorinostat ranked highest despite its confidence interval crossing 1.0, whereas bevacizumab plus lomustine had both a high ranking and a statistically significant effect. Accordingly, the latter provides more persuasive evidence of a PFS benefit. Nevertheless, because PFS is partly dependent on radiographic assessment, the observed benefit should be interpreted cautiously, particularly for bevacizumab-containing regimens in open-label trials without clearly reported blinded central review (6, 15, 16).

The PFS sensitivity analysis showed that the leading treatment rankings were largely unchanged after exclusion of Brown 2016 (35). Bevacizumab plus vorinostat, bevacizumab plus lomustine, bevacizumab plus re-irradiation, rindopepimut plus bevacizumab, and bevacizumab plus dasatinib remained the five highest-ranked treatments. The removal of cediranib plus gefitinib did not materially alter the relative ordering of the remaining interventions. These findings indicate that the principal PFS conclusions were reasonably robust to the exclusion of small trials.

In the OS analysis, none of the evaluated treatments demonstrated a statistically significant survival advantage over bevacizumab. However, bevacizumab should not be interpreted as a survival benchmark. Its use as the OS reference node reflected its central role as a comparator in the evidence network rather than established survival superiority; the network estimate for lomustine relative to bevacizumab was close to the null (HR = 1.05, 95% CI: 0.52–2.12). Rindopepimut plus bevacizumab had the lowest HR and ranked first according to the P-score, followed by regorafenib and tumor-treating fields (12, 13, 45). However, the confidence intervals for all OS comparisons crossed 1.0, and several were wide, indicating considerable uncertainty. Thus, the OS rankings should be interpreted as hypothesis-generating rather than as evidence that one regimen provides a confirmed survival benefit. Interpretation of the tumor-treating fields estimate requires additional caution. In Stupp 2012, the physician’s-choice active-chemotherapy control arm was mapped to the lomustine node to maintain network connectivity; because this comparator was not restricted to lomustine alone, the mapping may have introduced additional clinical heterogeneity and uncertainty into the tumor-treating fields estimate (13).

Including both REGOMA and the recurrent-disease cohort of GBM AGILE allowed regorafenib to be informed by two randomized comparisons against lomustine (14, 45). Regorafenib ranked second for OS but substantially lower for PFS and ORR. This pattern suggests that its relative position may differ across clinical outcomes and highlights the importance of avoiding conclusions based on a single endpoint. A treatment may influence survival without producing a correspondingly large radiographic response, particularly in recurrent glioblastoma, where response assessment may be affected by tumor heterogeneity, treatment-related imaging changes, and differences in subsequent therapy (16, 46).

The OS sensitivity analysis produced results that were nearly identical to those of the primary analysis. The five highest-ranked interventions remained rindopepimut plus bevacizumab, regorafenib, tumor-treating fields, bevacizumab plus vorinostat, and bevacizumab plus re-irradiation. Their P-scores changed only minimally after exclusion of the small Brown 2016 trial. This stability suggests that the relative OS rankings were not substantially driven by the excluded study. Nevertheless, the absence of statistically significant pairwise network estimates means that stable rankings should not be interpreted as definitive evidence of comparative superiority.

For ORR, no treatment significantly improved response relative to bevacizumab. Nivolumab was associated with a statistically significant reduction in the odds of objective response (10). This finding should be interpreted in the context of the different mechanisms and expected response patterns of anti-angiogenic and immunotherapeutic treatments. Bevacizumab can produce rapid radiographic changes by reducing vascular permeability and contrast enhancement, whereas radiographic responses to immunotherapy may be delayed or atypical (6, 15, 46). Consequently, ORR may favor treatments with a pronounced effect on imaging appearance and may not fully capture their long-term clinical benefit.

The risk-of-bias findings further support cautious interpretation of the PFS and ORR results. Several included studies were open label or did not clearly report blinded central radiographic assessment. Because both PFS and ORR depend on imaging interpretation, knowledge of treatment allocation may influence the classification or timing of response and progression. This concern is particularly relevant to bevacizumab-containing regimens, which can rapidly reduce contrast enhancement and edema through vascular effects without necessarily producing an equivalent reduction in viable tumor burden. Consequently, an apparent improvement in ORR or PFS may partly reflect radiographic pseudoresponse rather than durable antitumor activity (15, 16).

Differences in response-assessment criteria may have further contributed to this problem. Older studies frequently used the Macdonald criteria, whereas more recent trials generally applied RANO-based definitions, which incorporate non-enhancing disease and corticosteroid use more explicitly (15, 16, 25). The use of different criteria and variable blinding procedures may therefore have reduced the comparability of PFS and ORR across trials. In contrast, OS is less directly affected by radiographic interpretation and investigator judgment and may provide a more objective measure of clinical benefit. Nevertheless, OS may still be influenced by crossover, post-progression treatment, and differences in supportive care.

The primary ORR ranking placed ERC1671 combination plus bevacizumab first, followed by bevacizumab plus carboplatin, bevacizumab plus lomustine, rindopepimut plus bevacizumab, and galunisertib. However, the ERC1671-based regimen was supported by a trial with only four patients per arm and produced an extremely imprecise estimate. After small studies were excluded, the ERC1671-based regimen and cediranib plus gefitinib were removed from the network, whereas bevacizumab plus trebananib was retained because the Lee 2020 trial did not meet the small-trial exclusion criterion (35, 43, 44). Bevacizumab plus carboplatin then had the highest numerical P-score, followed by bevacizumab plus lomustine and rindopepimut plus bevacizumab. This change illustrates that very small studies can exert a disproportionate influence on P-score rankings, particularly when effect estimates are highly imprecise.

Despite the removal of these small-study nodes, the relative ordering of most retained ORR treatments remained broadly similar. Bevacizumab plus carboplatin, bevacizumab plus lomustine, rindopepimut plus bevacizumab, galunisertib, and VB-111 plus bevacizumab occupied the five highest positions in the sensitivity analysis. Nevertheless, none of these interventions significantly improved ORR relative to bevacizumab. The ORR findings therefore reinforce the need to distinguish between numerical treatment ranking and strength of evidence. The disappearance of the top-ranked ERC1671 regimen after exclusion of small studies demonstrates that rankings can be particularly fragile when networks include sparse comparisons.

Taken together, the results suggest that combination strategies containing bevacizumab may improve radiographic disease control and short-term progression-related outcomes in selected settings, although these effects may partly reflect vascular and imaging-related mechanisms and have not consistently translated into improved OS. Bevacizumab plus lomustine showed the most consistent PFS signal, whereas rindopepimut plus bevacizumab and regorafenib appeared promising for OS based on their relative rankings. However, these findings should not be interpreted as establishing a universal treatment hierarchy. Recurrent glioblastoma is biologically heterogeneous, and treatment effects may vary according to molecular characteristics, prior therapy, performance status, steroid use, and eligibility for reoperation or re-irradiation. In particular, biomarker-selected treatments may appear favorable within a restricted population but may not be generalizable to unselected patients.

Clinical and molecular heterogeneity should be considered when interpreting these findings. The included trials differed in treatment line, prior bevacizumab exposure, molecular classification, and biomarker-based eligibility. Patients treated at first recurrence may differ substantially from those receiving later-line therapy in performance status, tumor burden, treatment resistance, corticosteroid exposure, and eligibility for subsequent treatment. Prior bevacizumab exposure may represent a potential treatment-effect modifier because it could influence subsequent radiographic response and disease course (39, 47). Because these factors were not consistently reported and subgroup-specific comparative estimates were rarely available, their potential influence on the relative treatment effects could not be quantified.

Molecular heterogeneity may also limit the generalizability of the network estimates. Rindopepimut was evaluated in patients with EGFRvIII-positive tumors, and its favorable numerical ranking should therefore not be extrapolated to patients without EGFRvIII expression (12). Similarly, the activity of lomustine and other alkylating agents may vary according to MGMT promoter methylation status, but the included trials generally did not report comparative treatment effects separately for methylated and unmethylated tumors (48). Moreover, many older trials were conducted before routine IDH-based classification and did not report IDH status. Some enrolled tumors may therefore no longer meet the contemporary definition of IDH-wildtype glioblastoma, which limits the direct applicability of the pooled estimates to modern IDH-wildtype glioblastoma populations (1). The pooled estimates should therefore be interpreted as average trial-level effects across heterogeneous populations rather than as treatment effects applicable uniformly to all molecular subgroups. These differences are also relevant to the transitivity assumption underlying the network meta-analysis. Although the studies were broadly comparable with respect to the recurrent-glioblastoma setting and previous standard treatment, recurrence line, prior bevacizumab or lomustine exposure, performance-status eligibility, response criteria, and molecular selection were not fully comparable across all direct comparisons. The indirect estimates were therefore considered clinically plausible but subject to residual uncertainty, particularly for biomarker-selected treatment nodes and ORR comparisons involving anti-angiogenic therapies.

The findings of this network meta-analysis should be interpreted within current guideline frameworks rather than as establishing a stand-alone treatment hierarchy. Current EANO, ASCO–SNO, and SNO–EANO guidance consistently recognizes that no single salvage regimen is appropriate for all patients with recurrent glioblastoma (2, 49, 50). The updated ASCO–SNO guideline does not recommend for or against any specific therapeutic strategy because of the low certainty of the available evidence and recommends clinical-trial participation whenever feasible. EANO guidance similarly emphasizes individualized selection based on prior treatment, age, performance status, MGMT promoter methylation status, symptoms, and the pattern of disease progression.

A practical approach should begin with confirmation of true tumor progression and multidisciplinary assessment. Treatment decisions should take into account performance status, neurological function, corticosteroid requirement, lesion size and location, focal versus multifocal recurrence, interval from previous radiotherapy, prior systemic therapies, molecular profile, patient preferences, expected toxicity, and local treatment availability.

For patients with preserved performance status and a localized, surgically accessible recurrence, repeat resection may be considered when meaningful cytoreduction or symptom relief is achievable. Surgery may also provide tissue for updated molecular profiling, particularly when the original diagnosis preceded contemporary integrated molecular classification (51). For patients with a small focal recurrence and a sufficiently long interval from previous radiotherapy, re-irradiation may be considered after assessment of the previously delivered dose, target volume, and risk to surrounding normal brain tissue (11). Neither intervention should be selected solely on the basis of treatment rankings from the present network.

When systemic treatment is appropriate, enrolment in a well-designed clinical trial remains the preferred option. Outside a clinical trial, lomustine or another nitrosourea may be considered in patients with adequate performance status and bone-marrow reserve. Temozolomide rechallenge may be considered in selected patients with MGMT promoter-methylated tumors, particularly following a meaningful temozolomide-free interval and previous disease control (2, 48, 49). These decisions should account for prior hematological toxicity and cumulative exposure to alkylating agents.

In the present analysis, bevacizumab plus lomustine was associated with a statistically significant PFS benefit compared with lomustine, and this finding remained significant after exclusion of small trials. However, this result should not be interpreted as establishing broad superiority because the combination did not demonstrate a corresponding OS advantage (9). In addition, PFS effects involving bevacizumab may partly reflect reductions in vascular permeability, contrast enhancement, and edema rather than durable antitumor activity (15). Bevacizumab plus lomustine may therefore be considered as a disease-control option for selected patients who can tolerate its hematological and vascular toxicities, particularly when delaying progression or reducing disease-related symptoms is an important clinical objective. It should not be regarded as a universally preferred standard on the basis of the PFS finding alone.

Regorafenib requires particularly cautious interpretation. The REGOMA trial reported an OS benefit compared with lomustine, whereas the subsequent GBM AGILE platform trial did not confirm this advantage. In the present network, regorafenib had a favorable numerical OS P-score but did not demonstrate a statistically significant OS benefit compared with bevacizumab. Its use should therefore be individualized according to local guideline status, regulatory approval, availability, toxicity profile, prior anti-angiogenic treatment, and patient preference, rather than being selected solely on the basis of its ranking.

Rindopepimut plus bevacizumab also requires particularly restricted interpretation. Although this regimen showed the most favorable numerical OS estimate and the highest numerical OS P-score, the network estimate was not statistically significant compared with bevacizumab. More importantly, the supporting randomized evidence was derived from patients with EGFRvIII-positive recurrent glioblastoma. The observed signal therefore applies only to the biomarker-defined population studied and should not be extrapolated to EGFRvIII-negative patients or to an unselected recurrent glioblastoma population (12). In the absence of confirmatory randomized evidence, this finding should be regarded as exploratory rather than as a basis for routine treatment selection. Molecular features may further refine treatment selection, although comparative randomized evidence remains limited. Safety and patient-centered outcomes are also essential when translating these efficacy findings into clinical practice. The included trials reported adverse events using different categories, grading systems, and follow-up periods, while corticosteroid use, neurocognitive function, quality of life, and treatment burden were inconsistently assessed. These differences, together with the absence of extractable comparative data in several studies, precluded a reliable quantitative synthesis of these outcomes.

Bevacizumab-containing regimens may offer clinically relevant benefits through reductions in vascular permeability, peritumoral edema, neurological symptoms, and corticosteroid requirements (52). However, these symptomatic effects should not be equated with durable tumor control or improved OS and must be balanced against treatment-related toxicity. Alkylating agents and combination regimens may increase hematological toxicity and monitoring requirements (9), whereas device-based, radiation-based, and repeated intravenous treatments may impose substantial practical and logistical burdens. Because quality-of-life and neurocognitive outcomes were not consistently reported, the PFS, OS, ORR, and P-score results should not be interpreted as a complete measure of net clinical benefit. Treatment selection should therefore incorporate toxicity, symptom control, functional status, corticosteroid requirement, quality of life, treatment burden, patient preferences, and goals of care.

Rindopepimut-based findings apply specifically to patients with EGFRvIII-positive tumors and should not be extrapolated to an unselected recurrent glioblastoma population (12). MGMT promoter methylation may support consideration of alkylating-agent rechallenge (48, 53), whereas potentially actionable alterations, such as BRAF^V600E mutations or NTRK fusions, may justify molecularly matched therapy in an approved tumor-agnostic setting or within a clinical trial. Updated molecular testing is therefore particularly relevant when adequate tissue is available.

Patients with poor performance status, rapidly progressive multifocal disease, severe neurological impairment, or a low likelihood of meaningful benefit from further tumor-directed treatment should receive early supportive and palliative care. Symptom relief, corticosteroid optimization, seizure control, rehabilitation, advance-care planning, and caregiver support should be incorporated throughout the disease course rather than reserved for the final stages of illness (54).

Overall, the treatment algorithm can be summarized as follows: patients with a focal and surgically accessible recurrence may be considered for repeat resection; selected patients with a limited recurrence and an adequate interval from previous radiotherapy may be considered for re-irradiation; patients requiring systemic therapy should preferably enter a clinical trial, with lomustine, temozolomide rechallenge, bevacizumab, or selected combination therapy considered according to prior exposure, molecular status, symptoms, toxicity, and availability; and patients with poor performance status should be offered symptom-directed supportive care. This framework is intended to support multidisciplinary decision-making rather than to establish a definitive sequence of treatments.

This study has several strengths. It provides an updated quantitative synthesis of randomized evidence for recurrent or refractory glioblastoma and evaluates three clinically relevant outcomes. The use of a network meta-analytic framework enabled the simultaneous comparison of multiple interventions, including treatments that had not been directly compared in head-to-head trials. The updated analysis incorporated REGOMA, standardized treatment names across studies, and evaluated the influence of small studies through prespecified sensitivity analyses. The consistency of the main PFS and OS rankings after exclusion of small trials increases confidence in the robustness of these specific findings.

Several limitations should also be acknowledged. First, many treatment comparisons were informed by a single phase II trial, and several estimates had wide confidence intervals. The resulting P-scores may therefore imply a degree of precision that is not supported by the underlying evidence.

Second, substantial clinical and molecular heterogeneity existed across the included trials. Treatment line, prior bevacizumab exposure, MGMT promoter methylation status, IDH mutation status, and biomarker-based eligibility were incompletely or inconsistently reported. Subgroup-specific comparative treatment effects were rarely available, precluding reliable subgroup analyses or network meta-regression. In particular, IDH status was frequently unavailable in older trials, and some enrolled tumors might now be classified differently under contemporary diagnostic criteria. Consequently, the pooled estimates may not be fully applicable to modern populations defined specifically as IDH-wildtype glioblastoma. This heterogeneity may challenge the transitivity assumption underlying indirect comparisons and limits the applicability of pooled estimates to contemporary molecularly defined patient populations. Although the structured clinical assessment supported broad comparability at the overall disease-setting level, sparse and uneven reporting of potential effect modifiers prevented definitive confirmation of transitivity for every indirect comparison. Additionally, mapping the heterogeneous physician’s-choice chemotherapy arm in Stupp 2012 to the lomustine node may have introduced further clinical heterogeneity, particularly in estimates involving tumor-treating fields.

Third, some treatments were evaluated in highly selected populations. In particular, rindopepimut-based therapy was studied in patients with EGFRvIII-positive tumors, meaning that its relative effects and rankings should not be generalized to unselected patients. Furthermore, because EGFRvIII status was closely linked to the rindopepimut treatment node, aggregate-data meta-regression could not reliably distinguish a biomarker effect from a treatment effect. Similar concerns apply to MGMT promoter methylation and alkylating therapy, for which subgroup-specific comparative data were insufficient.

Fourth, the sensitivity analysis based on a threshold of 20 patients per arm was clinically useful but necessarily arbitrary. Exclusion of small trials reduced the influence of highly imprecise evidence but also removed unique treatment nodes, making it impossible to distinguish whether changes resulted from reduced small-study bias or from modification of the network structure.

Fifth, formal assessment of publication bias and small-study effects was limited by the small number of studies contributing to most comparisons.

Sixth, PFS and ORR may have been more vulnerable to performance and detection bias than OS. Several included studies were open label or did not clearly report blinded central radiographic assessment, and response definitions varied between the Macdonald and RANO criteria. These limitations are particularly relevant to bevacizumab-containing regimens because reductions in vascular permeability, contrast enhancement, and edema may produce radiographic pseudoresponse without an equivalent reduction in viable tumor burden (15, 16). Consequently, apparent improvements in PFS or ORR should not automatically be interpreted as evidence of durable tumor control or survival benefit. Although OS is less susceptible to radiographic assessment bias, it may still be influenced by crossover, subsequent therapy, and supportive care.

Seventh, adverse events and patient-centered outcomes, including corticosteroid-sparing effects, neurocognitive function, quality of life, and treatment burden, could not be quantitatively synthesized because their definitions, measurement instruments, assessment time points, and reporting formats varied substantially across trials. In several studies, these outcomes were absent or were not reported in an extractable comparative format. Consequently, the present comparisons based on PFS, OS, and ORR do not provide a complete assessment of the overall clinical benefit, toxicity, and treatment burden of each intervention. Treatment rankings should therefore be interpreted together with safety, symptom control, functional status, corticosteroid requirements, quality of life, patient preferences, and goals of care.

Eighth, inconsistency assessment was limited by the sparse network structure and the small number of closed loops. Although no statistically significant global inconsistency was detected, local node-splitting analyses and detailed direct-versus-indirect comparisons were not presented. Therefore, the absence of statistically significant global inconsistency should not be interpreted as definitive evidence that the transitivity and consistency assumptions were fully satisfied.

Ninth, the PROSPERO registration was completed on 18 May 2026, after the literature search had commenced, and was therefore not fully prospective. Although the outcomes and analytical methods were transparently reported and no eligible outcome was excluded according to the direction or statistical significance of the findings, this timing cannot completely eliminate the possibility of selective methodological or outcome reporting.

Finally, the OS evidence network was sparse and depended largely on central comparator nodes such as bevacizumab and lomustine. Many relative effects were therefore informed by limited direct evidence and should be interpreted as exploratory. Future randomized trials directly comparing active regimens across clinically relevant patient populations are needed to strengthen the connectivity and reliability of the evidence network.

In conclusion, bevacizumab plus lomustine provided the most consistent evidence of improved PFS, and this finding remained significant in the sensitivity analysis. No intervention demonstrated a statistically significant OS advantage over bevacizumab, although rindopepimut plus bevacizumab and regorafenib ranked favorably. For ORR, nivolumab was associated with lower odds of objective response than bevacizumab, while no treatment demonstrated a statistically significant improvement. Exclusion of small trials had little effect on the PFS and OS conclusions but materially changed the ORR hierarchy by removing the highly ranked ERC1671-based regimen. These findings emphasize that treatment rankings should always be interpreted alongside effect estimates, confidence intervals, sample sizes, and the precision and limitations of the underlying evidence.

## 5 Conclusion

This network meta-analysis provides an updated comparison of therapeutic strategies for recurrent or refractory glioblastoma. Bevacizumab plus lomustine showed the clearest evidence of improved PFS compared with lomustine and remained statistically significant after the exclusion of small trials. However, this finding was not accompanied by a statistically significant OS benefit and should not be interpreted as establishing broad treatment superiority. No intervention demonstrated a statistically significant OS advantage over bevacizumab. Although rindopepimut plus bevacizumab had a favorable numerical OS estimate, this finding was statistically non-significant and was derived from an EGFRvIII-positive population; it should therefore not be generalized to unselected patients with recurrent glioblastoma.

The sensitivity analyses indicated that the principal PFS and OS findings were generally robust. In contrast, the ORR ranking was more sensitive to the exclusion of small studies, particularly because the initially highest-ranked ERC1671 combination plus bevacizumab regimen was supported by a trial with only four patients per arm and was removed from the sensitivity analysis. These findings demonstrate that P-score rankings should not be interpreted independently of effect estimates, confidence intervals, sample sizes, network structure, and the precision of the underlying evidence.

Overall, bevacizumab plus lomustine showed the clearest evidence of improved PFS among the evaluated regimens. Large, well-designed randomized controlled trials with standardized outcome definitions and prospectively defined molecular subgroups are needed. Until such evidence becomes available, treatment decisions should be individualized through multidisciplinary assessment of performance status, recurrence pattern, prior treatment, corticosteroid requirement, molecular profile, treatment availability, and patient preferences.

## 6 Implications for practice

The findings of this network meta-analysis should be interpreted within existing guideline-based frameworks rather than as a stand-alone treatment ranking. In patients with suspected recurrent glioblastoma, the first step is to confirm true recurrence and distinguish it from pseudoprogression or treatment-related imaging change through multidisciplinary review of clinical status, imaging findings, treatment history, and, when appropriate, tissue or advanced imaging assessment.

After recurrence is confirmed, treatment selection should be individualized according to performance status, neurological symptoms, corticosteroid requirement, recurrence pattern, prior surgery and radiotherapy, previous systemic therapy, molecular profile, treatment goals, toxicity considerations, patient preference, drug availability, and eligibility for clinical trials. For patients with preserved performance status and localized surgically accessible recurrence, repeat resection may be considered when cytoreduction, symptom relief, or acquisition of tissue for updated molecular profiling is clinically relevant. Re-irradiation may be considered in selected patients with small focal recurrence, an adequate interval from prior radiotherapy, and acceptable normal-tissue constraints. In contrast, patients with poor performance status, extensive multifocal disease, or limited likelihood of benefit from further tumor-directed therapy should receive early supportive and palliative care.

For systemic therapy, enrollment in a well-designed clinical trial remains the preferred option whenever available. Outside a clinical trial, treatment choice should reflect prior exposure and the main therapeutic goal. Lomustine or another nitrosourea may be considered in eligible patients, whereas temozolomide rechallenge may be more appropriate for patients with MGMT promoter-methylated tumors and a sufficiently long treatment-free interval. Bevacizumab may be particularly useful when rapid reduction of edema, neurological symptoms, or corticosteroid dependence is needed and when the drug is locally available, although a consistent overall survival benefit has not been established.

The results of the present network meta-analysis may help contextualize these choices but should not replace individualized clinical judgment. Bevacizumab plus lomustine showed the clearest evidence of improved PFS and may be considered as a disease-control option for selected patients who can tolerate combination therapy. However, the absence of a corresponding OS benefit precludes interpretation of this regimen as a universally preferred standard. Rindopepimut plus bevacizumab showed a favorable numerical OS signal, but the supporting evidence was derived from an EGFRvIII-positive population and should not be generalized to unselected patients. Regorafenib should also be interpreted cautiously because evidence across randomized datasets has not been fully consistent. Overall, no single regimen can be broadly recommended as optimal for all patients with recurrent or refractory glioblastoma, and P-score rankings should not be used as stand-alone evidence for treatment selection.

## Data Availability

The original contributions presented in the study are included in the article/Supplementary material, further inquiries can be directed to the corresponding authors.
