# Supplementary material for: Comparative efficacy of therapeutic strategies for recurrent or refractory glioblastoma: a systematic review and network meta-analysis
Source: Front Neurol. 2026 Aug 20;17:1896062. doi: 10.3389/fneur.2026.1896062 (PMC13538010; doi:10.3389/fneur.2026.1896062)
Supplement: Supplementary file 1 [file Table_1.docx]

**Supplementary Methods.**

Pubmed(409):

(("Glioblastoma"[Mesh] OR "Glioma"[Mesh] OR glioblastoma[tiab] OR GBM[tiab] OR glioma*[tiab] OR "high-grade glioma"[tiab] OR "recurrent glioma"[tiab] OR "refractory glioma"[tiab]) AND ("Bevacizumab"[Mesh] OR bevacizumab[tiab] OR "Lomustine"[Mesh] OR lomustine[tiab] OR "Rindopepimut"[tiab] OR "Cediranib"[Mesh] OR cediranib[tiab] OR "Regorafenib"[Mesh] OR regorafenib[tiab] OR "Galunisertib"[tiab] OR "Enzastaurin"[tiab] OR "Vorinostat"[tiab] OR "Nivolumab"[Mesh] OR nivolumab[tiab] OR "Trebananib"[tiab] OR "Dasatinib"[Mesh] OR dasatinib[tiab] OR "Carboplatin"[Mesh] OR carboplatin[tiab])) AND (Randomized Controlled Trial[Publication Type] OR randomized[tiab] OR randomly[tiab] OR RCT[tiab]))

Web of science(402):

TS=(glioblastoma OR GBM OR glioma OR "high-grade glioma" OR "recurrent glioma" OR "refractory glioma")

AND

TS=(bevacizumab OR lomustine OR rindopepimut OR cediranib OR regorafenib OR galunisertib OR enzastaurin OR vorinostat OR nivolumab OR trebananib OR dasatinib OR carboplatin)

AND

TS=("randomized controlled trial" OR randomized OR randomly OR RCT)

Cochrane Library (108):

#1	MeSH descriptor: [Glioma] explode all trees

#2	high-grade glioma

#3	recurrent glioma

#4 #1 OR #2 OR #3

#5	MeSH descriptor: [Bevacizumab] explode all trees

#6	MeSH descriptor: [Lomustine] explode all trees

#7	lomustine

#8	Rindopepimut

#9	regorafenib

#10	Galunisertib

#11	Enzastaurin

#12	Vorinostat

#13	Trebananib

#14	dasatinib

#15	#5 OR #6 OR #7 OR #8 OR #9 OR #10 OR #11 OR #12 OR #13 OR #14

#16	#4 AND #15

**Supplementary Table S1. Global inconsistency assessment of the network meta-analysis.**

**Note.** PFS, progression-free survival; OS, overall survival; ORR, objective response rate. Global inconsistency was assessed separately for each outcome network. A P value > 0.05 was interpreted as showing no statistically significant evidence of global inconsistency. However, because the evidence networks were sparse and several comparisons were supported by limited direct evidence, the absence of statistically significant global inconsistency should not be interpreted as definitive proof of network consistency.

| Outcome | Global inconsistency P value | Interpretation |
| --- | --- | --- |
| PFS | 0.3127 | No statistically significant global inconsistency was detected. |
| OS | 0.3482 | No statistically significant global inconsistency was detected. |
| ORR | 0.8735 | No statistically significant global inconsistency was detected. |

**Supplementary Figure 1a.** Forest plot of the sensitivity analysis for progression-free survival.

**Supplementary Figure 1b.** P-score ranking of the sensitivity analysis for progression-free survival.

**Supplementary Figure 2a.** Forest plot of the sensitivity analysis for overall survival.

**Supplementary Figure 2b.** P-score ranking from the sensitivity analysis for overall survival.

**Supplementary Figure 3a.** Forest plot of the sensitivity analysis for objective response rate.

**Supplementary Figure 3b.** P-score ranking from the sensitivity analysis for objective response rate.
